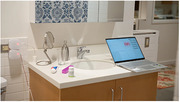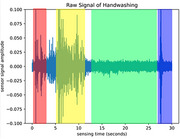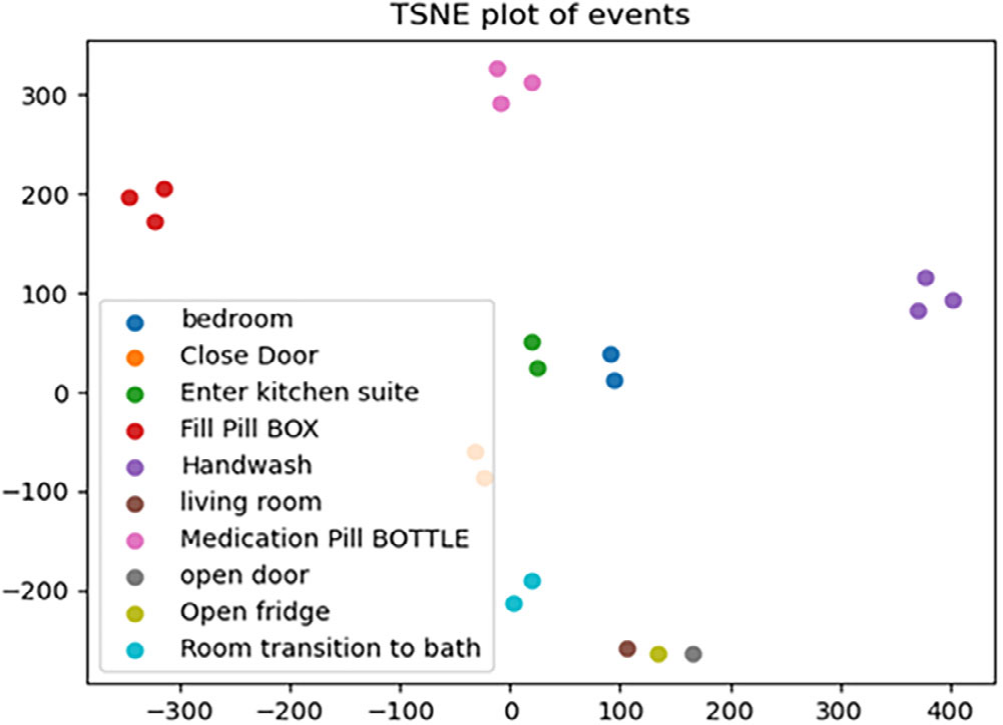# Vibration Sensing ‐ A Novel Approach to Detecting Activities of Daily Living

**DOI:** 10.1002/alz70856_104283

**Published:** 2025-12-24

**Authors:** Alyssa Weakley, Dong Lee, Blake Brown, Shijia Pan

**Affiliations:** ^1^ University of California, Davis School of Medicine, Sacramento, CA, USA; ^2^ University of California, Merced, Merced, CA, USA

## Abstract

**Background:**

One‐in‐four people with Alzheimer's disease (AD) desire to age‐in‐place alone. Due to cognitive and functional declines, adult children of individuals with AD often take on remote caregiving roles. Recent advancements in monitoring technology provide new opportunities for remote caregiving; however, limitations, particularly for detecting information about activities of daily living (ADLs) remain. To address this gap, we evaluated the potential of structural vibration technology to detect both coarse‐ (e.g., activity label ‐ hand washing) and fine‐ (e.g., specific step ‐ turn water on) grained ADL information.

**Method:**

Our novel approach to ADL detection uses geophone sensors and machine learning (ML) to recognize unique vibration patterns relating to a given activity. We collected controlled data in a 1‐bedroom simulation apartment testbed equipped with video cameras and sensors placed on the floor of the living room, bedroom, bathroom, and kitchen. As we were particularly interested in ADLs involving self‐care, an additional sensor was placed on the bathroom counter (see Figure 1). Ten young adult participants each completed 30 total sequences involving 3 (of possible 10) activities (e.g., wash hands, brush teeth, take medication). The dataset was manually labeled based on the video ground truth and applied to the vibration sensing data.

**Result:**

Pairing our ground truth labels with the vibration data, we were able to automatically detect activities including walking, talking, and medication taking in real time (Figure 1). Furthermore, we were able to identify specific fine‐grained actions of interest within a given activity (see Figure 2). Finally, we used Fast Fourier Transform t‐Stochastic Neighborhood Embedding for dimensionality reduction and visualization of the dataset. As shown in Figure 3, specific activities clustered closely together suggesting they produced similar vibration patterns over multiple sequences.

**Conclusion:**

We present an innovative approach to detecting ADLs using unobtrusive vibration sensing technology. Results suggest that we can detect both when any activity occurres and the specific actions that contribute to the activity. As such, vibration sensing is a promising tool for both intervention (e.g., prompting when error or omission occurs) and outcome purposes in ADRD clinical trial research.